# Adenosine‐Induced Coronary Steal Is Observed in Patients Presenting With ST‐Segment–Elevation Myocardial Infarction

**DOI:** 10.1161/JAHA.120.019899

**Published:** 2021-06-30

**Authors:** Muhammad Aetesam‐ur‐Rahman, Adam J. Brown, Catherine Jaworski, Joel P. Giblett, Tian X. Zhao, Denise M. Braganza, Sarah C. Clarke, Bobby S. K. Agrawal, Martin R. Bennett, Nick E. J. West, Stephen P. Hoole

**Affiliations:** ^1^ Department of Interventional Cardiology Royal Papworth Hospital Cambridge United Kingdom; ^2^ Division of Cardiovascular Medicine University of Cambridge Cambridge United Kingdom; ^3^ Department of Cardiology Monash University Melbourne Australia; ^4^ Department of Cardiology University Hospital Geelong Geelong Australia; ^5^ Department of Radiology Royal Papworth Hospital Cambridge United Kingdom

**Keywords:** adenosine, collateral circulation, microvascular dysfunction, ST‐segment–elevation myocardial infarction

## Abstract

**Background:**

Adenosine is used to treat no‐reflow in the infarct‐related artery (IRA) during ST‐segment–elevation myocardial infarction intervention. However, the physiological effect of adenosine in the IRA is variable. Coronary steal—a reduction of blood flow to the distal coronary bed—can occur in response to adenosine and this is facilitated by collaterals. We investigated the effects of adenosine on coronary flow reserve (CFR) in patients presenting with ST‐segment–elevation myocardial infarction to better understand the physiological mechanism underpinning the variable response to adenosine.

**Methods and Results:**

Pressure‐wire assessment of the IRA after percutaneous coronary intervention was performed in 93 patients presenting with ST‐segment–elevation myocardial infarction to calculate index of microvascular resistance, CFR, and collateral flow index by pressure. Modified collateral Rentrop grade to the IRA was recorded, as was microvascular obstruction by cardiac magnetic resonance imaging. Coronary steal (CFR <0.9), no change in flow (CFR=0.9–1.1), and hyperemic flow (CFR >1.1) after adenosine occurred in 19 (20%), 15 (16%), and 59 (63%) patients, respectively. Patients with coronary steal had higher modified Rentrop score to the IRA (1 [0, 1.75] versus 0 [0, 1], *P*<0.001) and a higher collateral flow index by pressure (0.25±0.10 versus 0.15±0.10, *P*=0.004) than the hyperemic group. The coronary steal group also had significantly higher index of microvascular resistance (61.68 [28.13, 87.04] versus 23.93 [14.67, 37.00], *P*=0.006) and had more disease (stenosis >50%) in the donor arteries (52.63% versus 22.03%, *P*=0.02) than the hyperemic group.

**Conclusions:**

Adenosine‐induced coronary steal may be responsible for a reduction in coronary flow reserve in a proportion of patients presenting with ST‐segment–elevation myocardial infarction.

**Registration:**

URL: https://www.clinicaltrials.gov; Unique identifier: NCT03145194. URL: https://www.isrctn.com; Unique identifier: ISRCTN3176727.

**Nonstandard Abbreviations and Acronyms**
BMR: basal microvascular resistance
CFI_P_
: collateral flow index by pressure
CFR: coronary flow reserve
FFR: fractional flow reserve
IMR: index of microvascular resistance
MVO: microvascular obstruction
PCI: percutaneous coronary intervention
RRR: resistive reserve ratio

**Clinical Perspective**

**What Is New?**

- The effect of adenosine on coronary physiology in the infarct‐related artery (IRA) during ST‐segment–elevation myocardial infarction is variable.
- We detect reduced coronary flow reserve after adenosine in up to 20% of patients with ST‐segment–elevation myocardial infarction—a phenomenon consistent with coronary steal.
- The coronary steal response in the IRA is associated with 3 variables: more profound microvascular dysfunction in the IRA measured by pressure wire and confirmed by cardiac magnetic resonance—a “closed,” nonresponding microvasculature, better collaterals to the IRA as evidenced by higher modified Rentrop score and collateral flow index by pressure >0.25, and higher angiographic prevalence of donor artery coronary artery disease (proximal vessel stenosis >50%).

**What Are the Clinical Implications?**

- The presence of these 3 variables may result in worsening of coronary flow in the IRA after adenosine during acute ST‐segment–elevation myocardial infarction and may be detrimental.

Adenosine is used to diagnose and treat periprocedural microvascular injury in the infarct‐related artery (IRA) territory during primary percutaneous intervention (PPCI).^1^, ^2^, ^3^ Early interventional studies have shown that adenosine infusion significantly reduced infarct size in patients with ST‐segment–elevation myocardial infarction (STEMI).^4^, ^5^ These initial positive results were challenged by another study that showed that infarct size actually increased after adenosine and that adenosine resulted in a higher major adverse cardiac event rate in patients with STEMI.^6^

Coronary steal is defined as a fall in coronary blood flow to a vascular region in favor of another supply area during hyperemia and is usually facilitated by well‐developed collaterals (horizontal steal).^7^, ^8^ Coronary steal is well described in stable severe coronary artery stenosis and chronic total occlusion and occurs when compensatory downstream microvascular vasodilation cannot respond further to hyperemic stimuli.^9^, ^10^, ^11^, ^12^ Remote coronary territories retain vasodilatory capacity, resulting in diversion of flow via collaterals away from the target vessel. It can be diagnosed by paradoxical reduction of coronary flow after administration of a hyperemic agent. Although physiologically plausible, this phenomenon has not been reported before in the acute STEMI setting.

We hypothesized that adenosine‐induced coronary steal would also occur in a subset of patients presenting with STEMI, and this may explain the variable effects of adenosine on coronary flow and clinical outcomes (Figure 1). We performed invasive pressure‐wire assessment of coronary flow in patients with STEMI after PPCI to (1) document changes in coronary flow reserve (CFR) that occur in response to adenosine, (2) identify incidence and frequency of coronary steal in response to adenosine, (3) elucidate and characterize any periprocedural parameters available at the time of intervention that could predict adenosine‐induced slow flow attributable to coronary steal, and (4) explore any magnetic resonance imaging–derived indices associated with coronary steal.

**Figure 1:**
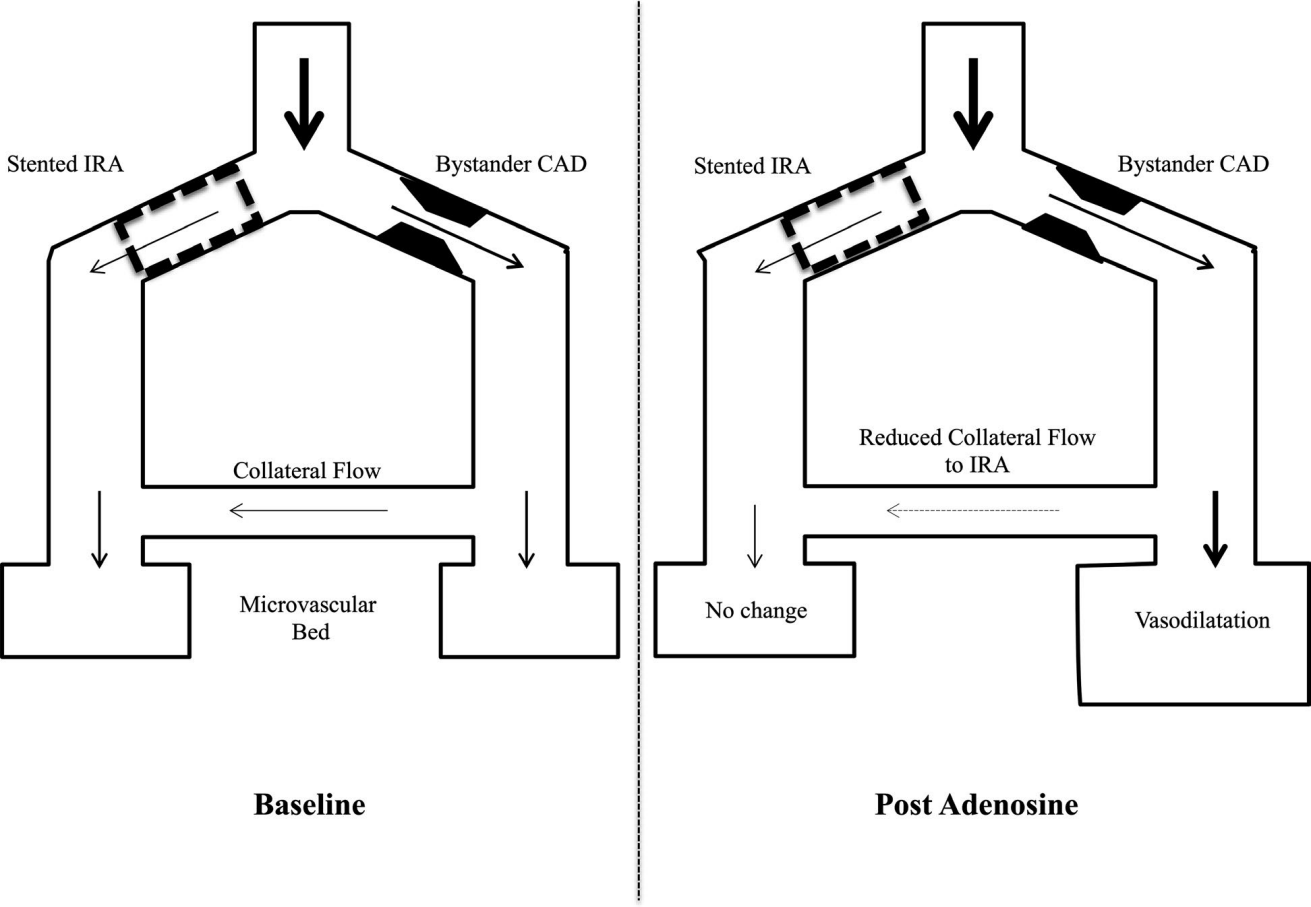
Schematic representation of coronary steal post‐PCI in patients presenting with STEMI. Fixed microvascular injury (“closed,” nonresponding microvasculature) in the stented infarct‐related artery (IRA) territory fails to respond to adenosine, whereas the non‐IRA‐related artery microcirculation retains the ability to vasodilate. An upstream stenosis in the donor artery will result in a pressure gradient favoring collateral‐dependent coronary steal—a fall in collateral flow during arteriolar vasodilatation to less than resting baseline levels. Quantification and direction of coronary flow is graphically depicted by size and darkness of arrow. CAD indicates coronary artery disease; PCI, percutaneous coronary intervention; and STEMI, ST‐segment–elevation myocardial infarction.

## Methods

Patients presenting to a single heart attack center with STEMI were recruited to this study. Detailed eligibility criteria and procedural details are given in Data S1.

### Angiographic Analysis

Pre‐ and post‐PPCI angiographic assessment of the IRA and bystander coronary arteries was performed to quantify TIMI (Thrombolysis in Myocardial Infarction) flow grading, TIMI myocardial blush grade, and evidence of collaterals supplying the IRA using the modified Rentrop score (Data S1).^13^, ^14^, ^15^, ^16^ Non‐IRA donor coronary arteries (providing collaterals to the IRA) were assessed for visual angiographic evidence of more than moderate stenosis (>50%). These arteries were expected to have adenosine‐induced reduction of distal coronary artery pressure (Pd).

### Invasive Coronary Physiological Assessment

**Figure 2:**
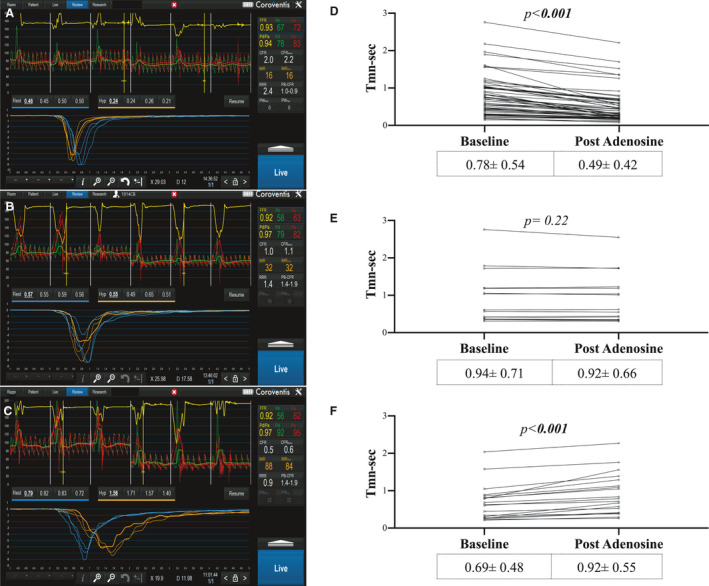
Stratification of patients by coronary flow reserve (CFR) derived by thermodilution transit time (Tmn). **A** and **B**, Hyperemic adenosine response, CFR >1.1, n=59; (**C** and **D**) No effect of adenosine, CFR=0.9 to 1.10, n=15; (**E** and **F**) Coronary steal after adenosine, CFR <0.90, n=19. Data are given as mean±SD, with *P*<0.05 given as bold.

Following successful stent implantation into the IRA, a Pressure wire X (Abbott Vascular, Santa Clara), connected wirelessly to Coroflow (Coroventis, Uppsala), was positioned in the distal third of the IRA. A 0.2‐mg bolus of intracoronary glyceryl trinitrate was administered, and once steady state of coronary hemodynamics was achieved, the baseline coronary pressures (aortic pressure [Pa] and distal wire pressures [Pd]) and flow velocity measurements were measured. The latter was derived from the reciprocal of mean transit time (Tmn) of an intracoronary injectate of room‐temperature saline (thermodilution technique) measured in triplicate, ensuring <10% variability.^16^, ^17^, ^18^, ^19^ These measurements were repeated following administration of adenosine at 140 μg/kg per minute (Figure 2A, 2C, and 2E). Coronary wedge pressure (Pw) was measured separately as Pd during the occlusive coronary balloon inflation during PCI. At the end of the procedure, the pressure wire was withdrawn to the coronary ostium to enable pressure‐drift correction of Pd, if necessary. Central venous pressure (Pv) was assumed to be 5 mm Hg in all the patients in this study.

These measurements enabled offline calculation of delta Pa (Pa_baseline_−Pa_hyperemia_), delta Pd (Pd_baseline_−Pd_hyperemia_), basal microvascular resistance (BMR=Pa×Tmn×((Pd−Pw)/(Pa−Pw))_baseline_) and index of microvascular resistance (IMR=Pa×Tmn×((Pd−Pw)/(Pa−Pw))_hyperemia_), both corrected for collaterals, fractional flow reserve (FFR=(Pd)/(Pa)_hyperemia_), coronary flow reserve (CFR=(Tmn)_baseline_/(Tmn)_hyperemia_), and collateral flow index by pressure (CFI_P_=(Pw−Pv)/(Pa−Pv)_baseline_) and coronary resistive reserve ratio (RRR=BMR/IMR), as previously described and validated.^20^, ^21^, ^22^ A cut‐off value of CFI_P_ of 0.25 was used to identify patients with physiological evidence of good collaterals.^23^, ^24^, ^25^ Similarly, an IMR >40 was used to identify patients with STEMI with significant microvascular injury.^26^

### Study Groups

An arbitrary CFR cut‐off of <0.90 was used to define a reduction of coronary flow and therefore evidence of coronary steal. Patients were stratified into 3 groups according to the adenosine response: hyperemic (CFR >1.1), no effect (CFR 0.9–1.1), and coronary steal (CFR <0.9).

### Validation Data

We further performed a validation analysis using a pressure‐derived index of coronary vasodilatory capacity, relative resistive index (RRR), and magnetic resonance imaging parameters to confirm our findings.

### Cardiac Magnetic Resonance Imaging Analysis

Cardiac magnetic resonance (CMR) studies were performed within 24 to 72 hours of PPCI. Late gadolinium evidence of microvascular obstruction (MVO) was recorded as a binary measurement as well as quantified as percentage of left ventricle mass as previously described^27^ (Data S1).

### Statistical Analysis

The authors declare that all supporting data are available within the article and its online supplementary files.

Data are given as mean±SD or median (Q1, Q3), and n (%) unless otherwise stated. Comparisons were made for any significant differences by unpaired *t* test, 1‐way ANOVA, or Kruskal–Wallis test, where appropriate. Following identification of significant differences, a post hoc analysis with Holm‐Šídák multiple comparison test or Dunn's multiple comparison test were used using GraphPad Prism version 8.1.2 (227) (GraphPad Software, La Jolla, CA). Receiver operating characteristics curves were assessed to measure area under the curve for bystander coronary artery disease (CAD), high IMR, and high CFI_P_ to predict slow flow because of coronary steal with 95% CI. We also performed multivariate logistic regression analysis of baseline characteristic covariates and their relationship with coronary steal on IBM SPSS Statistics for Macintosh, version 27 (IBM Corp., Armonk, NY). A 2‐sided value of *P*<0.05 was deemed significant. Authors had full access to and take full responsibility for the integrity of the data.

The local research ethics committee approved study NCT03145194 and ISRCTN31767278 protocols (REC reference 15/EE/0032 and 08/H0306/49, respectively). Both of the trials conformed to the principles outlined in the Declaration of Helsinki and were approved by our institution review committee. All subjects gave informed consent.

## Results

### Study Population

**Figure 3:**
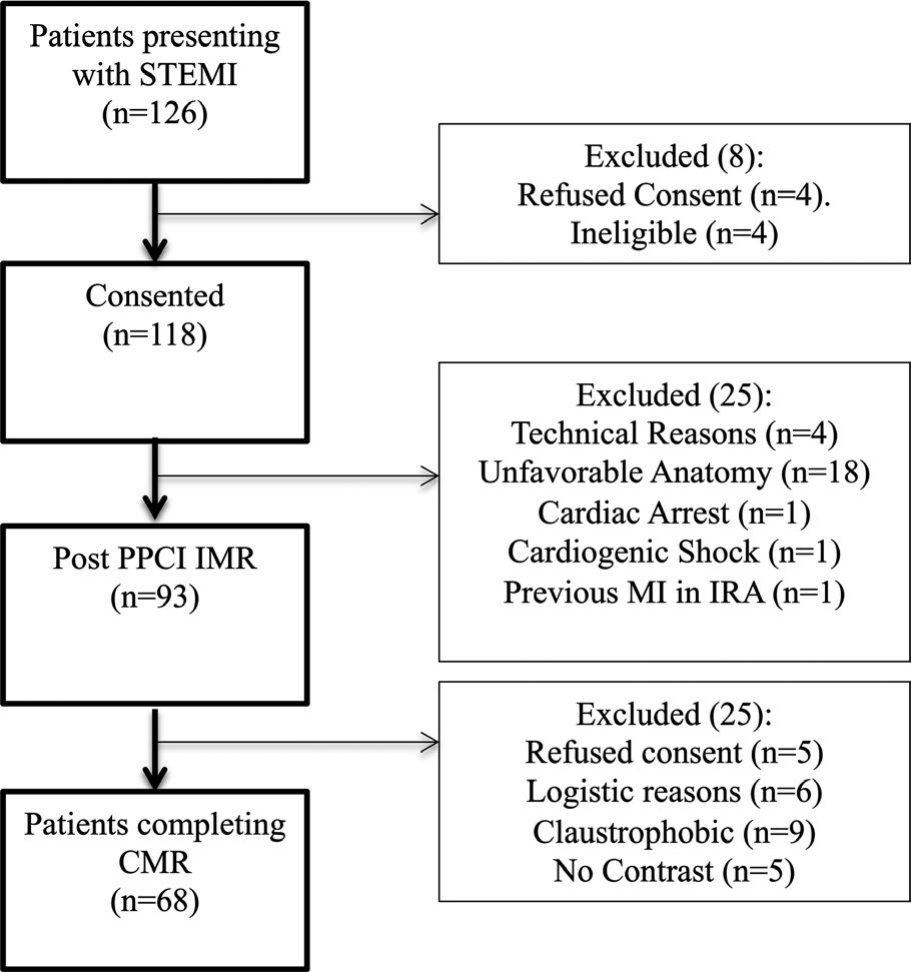
Recruitment details of study patients. CMR indicates cardiac magnetic resonance; IMR, index of microcirculatory resistance; IRA, infarct‐related artery; MI, myocardial infarction; PPCI, primary percutaneous coronary intervention; and STEMI, ST‐segment–elevation myocardial infarction.

One hundred twenty‐six patients presenting with STEMI were approached, 118 patients agreed to participate, and 93 (79%) patients successfully had the primary outcome measured during PPCI. The reasons for patients' exclusion from the study during PPCI are summarized in Figure 3. Sixty‐eight patients out of successfully recruited patients (73%) subsequently completed an inpatient CMR study (Figure 3).

### Incidence of Adenosine‐Induced Coronary Steal

Invasive evidence of hyperemic response to adenosine (CFR >1.1) was found in 59 (63%) patients (Figure 2A and 2B). Adenosine infusion did not enhance coronary flow velocity in a significant proportion of patients, including patients with no effect of adenosine on coronary flow reserve (CFR=0.9–1.1), n=15 (16%) (Figure 2C and 2D) and a further subset of patients, n=19 (20%), showing a paradoxical adenosine‐induced coronary steal (CFR <0.9) (Figure 2E and 2F).

### Baseline and Angiographic Characteristics

The majority of the patients studied were male and presented with anterior STEMI. We found no significant differences in the baseline demographics among the 3 groups, with a high prevalence of hypertension, diabetes mellitus, and history of smoking across all the 3 groups (Table 1). Pre‐PCI median TIMI flow was significantly lower, the modified Rentrop grade of collaterals supplying the IRA was higher, and the non‐IRA donor vessel was more likely to have visual CAD (stenosis >50%) in the coronary steal group compared with patients with hyperemic response to adenosine (Table 2). There was no relationship identified on multivariate analysis between any of the baseline characteristics and the incidence of coronary steal (Data S1).

**Table 1:** Baseline Characteristics

	Coronary Steal (n=19)	No Effect (n=15)	Hyperemic (n=59)	*P* Value
Age, y	72.59 (9.56)	73.80 (10.45)	67.63 (11.30)	0.12*
Male patients, n (%)	16 (84.21)	10 (66.67)	50 (84.74)	0.28*
Diabetes mellitus, n (%)	5 (26.31)	3 (20.00)	21 (35.59)	0.78*
Hypertension, n (%)	11 (57.89)	5 (33.33)	31 (52.54)	0.13*
Smoker, n (%)	5 (26.31)	8 (53.33)	22 (37.28)	0.29*
Hypercholesterolemia, n (%)	3 (15.79)	5 (33.33)	9 (15.25)	0.44*
Prior statin use, n (%)	5 (26.31)	5 (33.33)	16 (27.11)	0.67*
Previous MI, n (%)	3 (15.79)	2 (13.33)	4 (6.77)	0.75*

Baseline characteristics are expressed as mean (SD) or n (%). Comparison is made between all of the 3 study groups: coronary steal (CFR <0.9); hyperemic response (CFR >1.1), and patients with no effect of adenosine (CFR=0.9–1.1). CFR indicates coronary flow reserve; and MI, myocardial infarction.

* *P*<0.05.

**Table 2:** Angiographic Characteristics of Patients

	Coronary Steal (n=19)	No Effect (n=15)	Hyperemic (n=59)	*P* Value
LAD, n (%)	13 (68.42)	9 (60.00)	34 (57.63)	0.70
RCA, n (%)	4 (21.05)	3 (20.00)	15 (25.42)	0.87
LCX, n (%)	2 (10.53)	3 (20.00)	10 (16.95)	0.73
Bystander CAD, n (%)	10 (52.63)	7 (46.67)	13 (22.03)	0.02*
Modified Rentrop collateral score	1 (0, 2)	1 (0, 1)	0 (0, 0)	<0.001*
Pre‐PCI				
TIMI flow	0 (0, 0)	0 (0, 0)	2 (0, 2.5)	0.01*
Post‐PCI				
TIMI flow	3 (3, 3)	3 (3, 3)	3 (3, 3)	0.56
TMBG	2 (2, 2.5)	2.5 (2, 3)	3 (2, 3)	0.57

Angiographic features are expressed as n (%) and median (Q1, Q3). All the variables are compared among the 3 groups. CAD indicates coronary artery disease; LAD, left anterior descending artery; LCX, left circumflex artery; PCI, percutaneous coronary intervention; RCA, right coronary artery; TIMI, thrombolysis in myocardial infarction; and TMBG, TIMI myocardial blush grade.

* *P*<0.05.

### Hemodynamic Assessment

#### Coronary Pressure

There were no significant differences in the adenosine‐induced pressure drop in the IRA (delta Pa and delta Pd) across all the 3 groups of patients (Table 3).

**Table 3:** Invasive Hemodynamic Findings

	Coronary Steal (n=19)	No Effect (n=15)	Hyperemic (n=59)	*P* Value
Baseline				
Tmn baseline, s	0.69 (0.48)	0.94 (0.71)	0.78 (0.54)	0.43
Pa baseline, mm Hg	91.47 (18.63)	82.33 (19.16)	99.32 (16.06)	0.003*
Pd baseline, mm Hg	87.32 (19)	79.26 (18.75)	95.15 (15.75)	0.004*
BMR, mm Hg·s	52.86 (22.57, 78.09)	47.08 (27.90, 134.80)	56.13 (28.07, 91.71)	0.49
Pd/Pa	0.95 (0.04)	0.96 (0.05)	0.95 (0.06)	0.81
Hyperemia				
Tmn hyp, s	0.92 (0.55)	0.92 (0.66)	0.49 (0.42)	<0.001*
Pa hyp, mm Hg	79.57 (24.33)	67.73 (16.78)	88.84 (15.87)	<0.001*
Pd hyp, mm Hg	75.78 (23.64)	64.66 (16.44)	82.52 (15.14)	0.002*
FFR	0.95 (0.04)	0.95 (0.07)	0.93 (0.06)	0.39
IMR, mm Hg·s	61.68 (28.13, 87.04)	31.35 (21.12, 97.91)	23.93 (14.67, 37.00)	0.006*
RRR	0.85 (0.21)	1.22 (0.26)	2.03 (0.80)	<0.001*
CFR	0.74 (0.14)	1.01 (0.04)	1.78 (0.64)	<0.001*
Delta Pa, mm Hg	12.00 (6.00, 18.00)	11.00 (3.00, 26.00)	11.00 (3.00, 16.00)	0.59
Delta Pd, mm Hg	11.00 (7.00, 21.00)	11.00 (2.00, 24.00)	13.00 (6.00, 18.00)	0.88
Collateral circulation				
Pw, mm Hg	25.53 (8.94)	15.13 (11.01)	19.47 (10.53)	0.01*
CFI_P_	0.25 (0.11)	0.18 (0.12)	0.15 (0.11)	0.02*
CFI_P_ >0.25, n (%)	12 (63.16)	4 (26.67)	15 (25.42)	0.02*

Results expressed as mean (SD), median (Q1, Q3), and n (%). BMR indicates basal microcirculatory resistance=Pa×Tmn_baseline_×((Pd−Pw)/(Pa−Pw)); CFI_P_, collateral flow index by pressure=(Pw−Pv)/(Pa−Pv)_baseline_; CFR, coronary flow reserve=Tmn_baseline_/Tmn _hyperemic_; FFR, fractional flow reserve=Pd/Pa_hyperemic_; IMR, index of microcirculatory resistance=Pa×Tmn_hyperemic_×((Pd−Pw)/(Pa−Pw)); Pa, aortic pressure; Pd, distal coronary pressure; Pw, coronary wedge pressure; RRR, coronary resistive reserve ratio=BMR/IMR; and Tmn, transit time.

* *P*<0.05.

#### Microvascular Resistance

There were no significant differences in the mean BMR among the 3 groups, but IMR was significantly higher in patients with coronary steal than in patients with a hyperemic response to adenosine: 61.68 (28.13, 87.04) versus 23.93 (14.67, 37.00), *P*=0.006. Mean RRR was lower in the coronary steal group and in patients with no effect of adenosine on the flow as compared with patients exhibiting a hyperemic response (Table 3).

#### Coronary Flow Velocity Assessment

Although mean Tmn was not different across the 3 groups at baseline, it became significantly higher in patients in the coronary steal group as compared with the patients with a hyperemic response after adenosine infusion (Figure 2, Table 3).

#### Collateral Flow

There was higher mean CFI_P_ in the patients exhibiting coronary steal as compared with the hyperemic group. The hemodynamic evidence of well‐developed collaterals (CFI_P_ >0.25) was more frequently observed in the coronary steal group, and the mean Pw was also higher (Table 3).

### CMR Results

CMR‐derived mean left ventricular volume and mass were not different in the 3 groups (Table 4). CMR evidence of MVO was more frequent in patients with coronary steal as compared with patients in the hyperemic group, but the mean infarct size was not different between the 2 groups.

**Table 4:** Cardiac MRI Indices

	Coronary Steal (n=14)	No Effect (n=10)	Hyperemic (n=44)	*P* Value
EDV, mL	164.86 (25.31)	156.91 (33.86)	165.84 (37.59)	0.73
ESV, mL	89.21 (20.51)	86.95 (33.44)	89.14 (32.06)	1.00
LVEF, %	46.32 (8.65)	44.27 (14.45)	46.34 (11.18)	0.55
LV mass, g	127.53 (41.18)	112.45 (24.63)	119.29 (30.56)	0.61
Infarct size, g	23.11 (15.19)	19.25 (14.24)	15.92 (9.36)	0.12
Infarct size (% of LV mass)	17.85 (2.99)	16.42 (3.14)	16.95 (3.30)	0.70
Incidence of MVO, n (%)	12 (85.71)	4 (40.00)	20 (45.45)	0.02*
MVO mass, g	3.63 (3.83)	3.28 (4.51)	0.99 (2)	0.01*
MVO (% of LV mass)	2.58 (3.50)	2.40 (3.23)	0.83 (1.91)	0.01*

Results expressed as mean (SD) and n (%). EDV indicates end diastolic volume; ESV, end systolic volume; LV, left ventricle; LVEF, left ventricular ejection fraction; MRI, magnetic resonance imaging; and MVO, microvascular obstruction.

* *P*<0.05.

### Validation by RRR

IMR, P_W_, and CFI_P_ were higher in patients presenting with STEMI with RRR <1 as compared with patients with RRR >1. Similarly, magnetic resonance imaging indices followed similar trends, with higher incidence of MVO and larger area of left ventricle subtended by MVO in patients with RRR <1 (Table 5).

**Table 5:** Validation of Parameters by RRR

	RRR <1 (n=13)	RRR >1 (n=80)	*P* Value
Hemodynamic data			
Baseline			
Tmn baseline, s	0.69 (0.27, 0.85)	0.64 (0.36, 1.04)	0.38
Pa baseline, mm Hg	94.46 (18.08)	95.06 (18.20)	0.91
Pd baseline, mm Hg	82.46 (24.31)	77.59 (17.34)	0.91
BMR, mm Hg·s	64.27 (22.75, 78.94)	53.55 (30.73, 100.40)	0.45
Pd/Pa	0.96 (0.94, 0.98)	0.96 (0.93, 0.99)	0.74
Hyperemia			
Tmn hyp, s	0.84 (0.47, 1.34)	0.42 (0.24, 0.71)	0.007*
Pa hyp, mm Hg	85.54 (25.42)	83.23 (18.23)	0.69
Pd hyp, mm Hg	82.46 (24.31)	77.59 (17.34)	0.37
FFR	0.95 (0.94, 0.99)	0.94 (0.91, 0.98)	0.08
IMR, mm Hg·s	71.76 (33.86, 98.22)	29.35 (17.47, 56.03)	0.006*
CFR	0.72 (0.56, 0.79)	1.43 (1.08, 1.75)	<0.0001*
Collateral circulation			
Pw, mm Hg	25.00 (19.50, 34.00)	21.50 (10.50, 28.00)	0.03*
CFI_P_	0.29 (0.15, 0.38)	0.19 (0.07, 0.29)	0.03*
MRI indices			
EDV, mL	166.90 (29.78)	165.40 (35.83)	0.91
LV mass, g	130.90 (40.91)	119.60 (32.28)	0.36
LVEF, %	45.35 (8.75)	47.03 (10.88)	0.66
Incidence of MVO, n (%)	9 (100)	23 (46.94)	0.03*
MVO (% of LV mass)	3.03 (0.51, 7.10)	0 (0, 1.20)	0.001*

Comparison of hemodynamic and MRI indices using cut‐off value of RRR of 1. Results expressed as mean (SD), median (Q1, Q3), and n (%). BMR indicates basal microcirculatory resistance=Pa×Tmn_baseline_×((Pd−Pw)/(Pa−Pw)); CFI_P_, collateral flow index by pressure=(Pw−Pv)/(Pa−Pv)_baseline_; CFR, coronary flow reserve=Tmn_baseline_/Tmn_hyperemic_; EDV, end diastolic volume; FFR, fractional flow reserve=Pd/Pa_hyperemic_; IMR, index of microcirculatory resistance=Pa×Tmn_hyperemic_×((Pd−Pw)/(Pa−Pw)); LV, left ventricle; LVEF, left ventricular ejection fraction; MRI, magnetic resonance imaging; MVO, microvascular obstruction; Pa, aortic pressure; Pd, distal coronary pressure; Pw, coronary wedge pressure; RRR, coronary resistive reserve ratio=BMR/IMR; and Tmn, transit time.

* *P*<0.05.

### Prediction of Coronary Steal in Catheterization Lab

**Figure 4:**
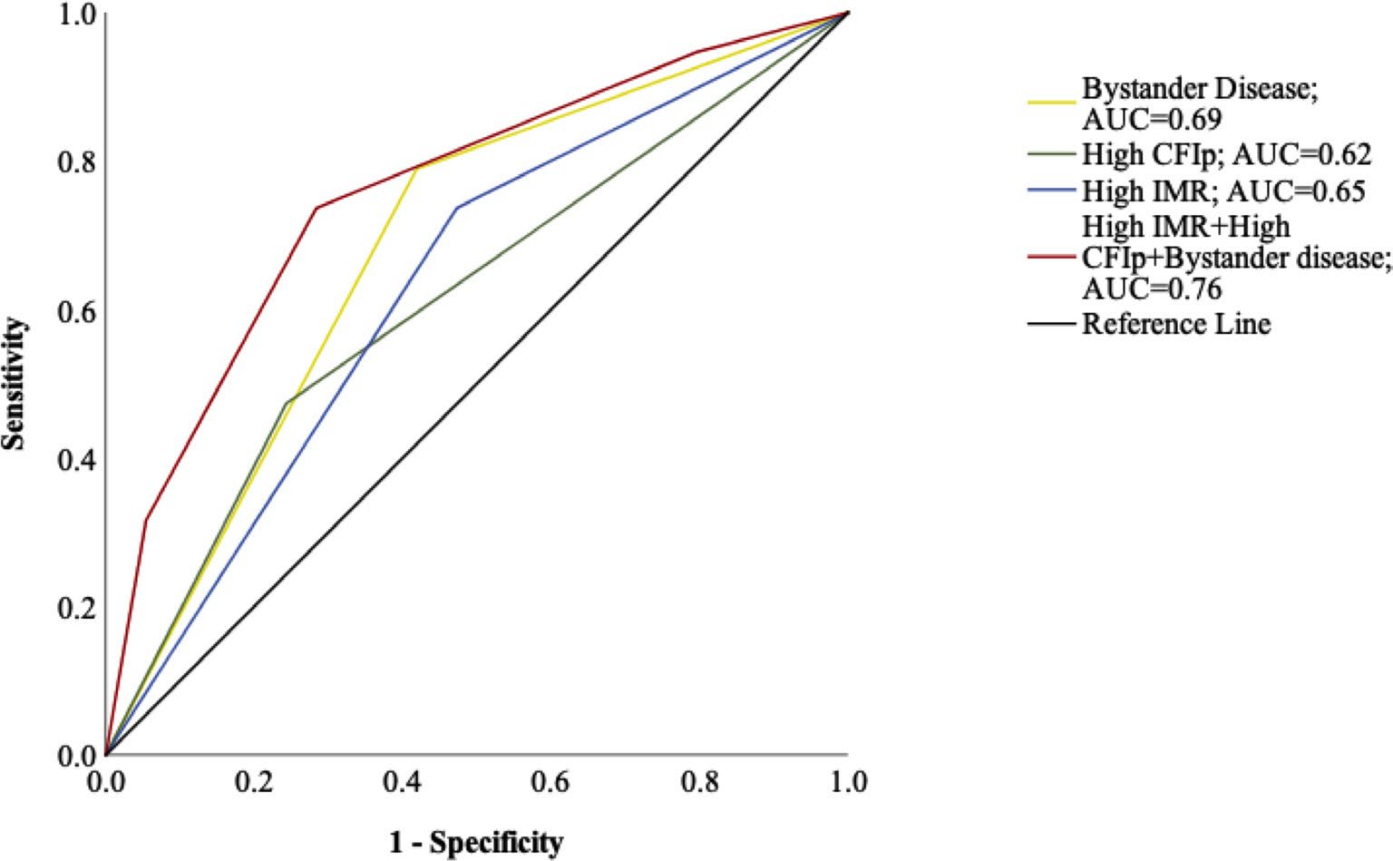
Receiver operator curves of the parameters (alone and in combination) associated with slow flow response to adenosine and coronary steal. AUC indicates area under the curve; CFI_p_, collateral flow index by pressure; and IMR, index of microcirculatory resistance.

Identification of non‐IRA bystander CAD in the donor vessel can predict presence of coronary steal in the IRA with an area under the curve of 0.68 (95% CI, 0.56–0.81; *P*=0.01). Presence of high IMR in the IRA was also associated with an area under the curve of 0.65 (95% CI, 0.51–0.80; *P*=0.03). The strongest prediction model was found in the presence of all 3 factors associated with coronary steal (ie, high CFI_P_, bystander CAD and high IMR, area under the curve of 0.78) (95% CI, 0.66–0.90), *P*<0.001 (Figure 4).

## Discussion

The main findings of this study are as follows: (1) coronary flow response to adenosine is variable in patients presenting with STEMI; (2) 20% of patients with STEMI have evidence of paradoxical slow flow and coronary steal in response to adenosine in the IRA; (3) coronary steal response is associated with more profound microvascular dysfunction measured by pressure wire and confirmed by CMR, better collaterals to the IRA as evidenced by higher modified Rentrop score and CFI_P_, and higher angiographic prevalence of donor artery CAD; and (4) detection of significant microvascular injury (IMR >40), physiological evidence of good collaterals (CFI_P_ >0.25), and non‐IRA CAD can predict coronary steal in the IRA.

We describe for the first time the phenomenon of coronary steal in response to adenosine in patients with STEMI. This may explain why the presence of collaterals to the IRA does not universally portend a better outcome in STEMI.^28^, ^29^ We have confirmed that the combination of 3 well‐described prerequisites for coronary steal to occur in patients with stable severe stenosis and chronic total occlusions, also predict coronary steal in the IRA after PPCI.^12^, ^30^, ^31^ First, reduction of donor artery pressure (Pd) proximal to the origin of collaterals because of an upstream stenosis is necessary. Second, a good collateral supply to the IRA is needed for horizontal coronary steal to occur. Third, in the original description, maximal vasodilatation of the recipient artery microcirculation is also stipulated, so that it lacks any further vasodilatory reserve (“open,” nonresponding microvasculature) relative to the donor artery, which retains vasodilatory capacity.^32^ All 3 prerequisites were observed in the coronary steal STEMI cohort, including fixed microvascular injury (“closed,” nonresponding microvasculature), confirmed by higher IMR and more MVO in those unable to respond to adenosine (Figure 1). Microvascular injury results in suboptimal procedural success and is associated with worse prognosis in patients presenting with STEMI.^33^, ^34^

The presence of collateral‐dependent coronary steal in patients with stable angina and single‐vessel disease is associated with a higher extent of ischemia and worse symptom burden both pre‐ and post‐PCI.^35^, ^36^, ^37^ Although the presence of collaterals is generally considered a marker of better prognosis, their value is controversial in acute myocardial infarction treated with PCI, with disparate effects on infarct size, left ventricle remodeling, and long‐term outcomes in different trials.^28^, ^29^, ^38^, ^39^, ^40^, ^41^ The presence of good collaterals at the time of PCI for acute coronary syndrome is particularly associated with higher risk of target vessel re‐occlusion, in‐stent restenosis, and mortality.^29^, ^38^, ^42^ This could be because of collateral‐dependent coronary steal occurring in response to endogenous vasodilators worsening coronary flow and possibly exacerbating microvascular injury, resulting in poorer myocardial function and worse long‐term outcome in this STEMI subgroup of patients.^26^, ^43^

Furthermore, the presence of an upstream disease in the donor artery likely reduces the distal coronary pressure in the donor vessel relative to the distal vessel of the recipient (IRA), resulting in a reduction or in some instances even a reversal of the coronary collateral pressure gradient and collateral flow, exacerbating coronary steal (Figure 1). Complete revascularization performed during PPCI in patients presenting with STEMI is associated with significant reductions in both total mortality and recurrent ischemic events.^44^, ^45^, ^46^ Complete revascularization not only improves global myocardial perfusion, but by removing upstream disease in the non‐IRA, it may also reduce an incremental ischemic insult in the IRA from coronary steal in the subset of patients with STEMI susceptible to paradoxical slow flow caused by coronary steal.

### Study Limitations

There are several limitations of our proof‐of‐concept study that need addressing. First, our data are retrospective from a relatively small cohort of predominantly male patients with STEMI and require further confirmation with a larger prospective study. However, patients with STEMI were recruited consecutively and studied in detail and we believe our findings remain valid and are hypothesis generating. Second, we did not perform simultaneous hemodynamic assessment of the non‐IRA donor coronary artery because of logistic reasons and cannot validate the coronary steal hypothesis presented by invasively calculating collateral coronary flow to explain our IRA observations. However, all 3 prerequisites required for horizontal steal were prevalent in our study's coronary steal group. Third, we did not invasively measure Pv in our study patients, again for logistic reasons. Patients who were in heart failure or cardiogenic shock were not recruited and there were no differences in CMR derived left ventricular ejection fraction between the groups. Fourth, we administered intravenous adenosine at 1 time interval, post‐PCI. Although we cannot extrapolate the data to predict effects of adenosine administered at different doses or timings, given that our hemodynamic data are consistent with our CMR findings, we believe that changing the administration parameters is unlikely to alter our conclusions. Fifth, we used a surrogate for absolute coronary flow: coronary flow velocity, measured as coronary transit time using the thermodilution technique at a stable thermistor position. This technique is well established, validated, and comparable with other clinically available methods. Nevertheless, we further validated our findings using RRR and found similar trends in the parameters associated with coronary steal in our CFR‐defined study groups. Sixth, CFI_p_ may not accurately quantify collaterals in STEMI and may simply reflect microvascular injury.^47^ However, semiquantification of collaterals by modified Rentrop score was also significantly higher in the adenosine‐induced slow flow/coronary steal group than the hyperemic group. Finally, adenosine‐induced slow flow may have an alternative explanation; by exhausting IRA vasodilatory capacity, pressure becomes the main driver of flow, so that an enhanced drop in the coronary pressure gradient caused by adenosine in those with larger infarcts and significant microvascular dysfunction could cause slow flow. In addition, hypotension could elicit a sympathetic baroreflex and catecholamine‐induced microvascular vasospasm. However, we can dismiss these potential mechanisms by observing that there were no significant differences in adenosine‐induced epicardial pressure drop in the IRA across the 3 groups, as measured by delta Pa and delta Pd, making coronary steal the most likely explanation.

## Conclusions

Coronary steal phenomena in response to adenosine are seen in a significant proportion of patients with STEMI. Patients with significant microvascular injury in an IRA territory that is subtended by collaterals from a donor vessel with upstream stenosis appear to be particularly susceptible. Results from this proof‐of‐concept study are hypothesis generating and should be confirmed by further larger studies exploring the physiological effects of adenosine in patients presenting with STEMI.

## Sources of Funding

This study was funded by the NIHR Cambridge Biomedical Research Centre and a research grant from AstraZeneca.

## Disclosures

At the time of study, authors had no conflicts of interest to declare. West has been appointed as CMO of Abbott Vascular since completion of this study.

## Supporting information

Data S1
