# Supplementary material for: Adenosine‐Induced Coronary Steal Is Observed in Patients Presenting With ST‐Segment–Elevation Myocardial Infarction
Source: J Am Heart Assoc. 2021 Jun 30;10(13):e019899. doi: 10.1161/JAHA.120.019899 (PMC8403291; doi:10.1161/JAHA.120.019899)
Supplement: Supplementary file 1 — Data S1 [file JAH3-10-e019899-s001.pdf]

SUPPLEMENTAL MATERIAL

Data S1.

Supplemental Methods and Results

Eligibility Criteria

Inclusion criteria: (i) age at enrollment >18 and <90 years (ii) ECG-confirmed STEMI (ST elevation ≥2 mm in ≥2 contiguous chest leads or ≥1 mm in ≥2 contiguous limb leads) or new left bundle branch block (LBBB) (iii) chest pain for <24h and (iv) ability to give informed consent.

Exclusion criteria: (i) cardiogenic shock (ii) previous infarct in the IRA territory (iii) unfavourable coronary anatomy (left main occlusion or distal vessel occlusion or grafts) (iv) severe asthma or bradycardia precluding use of adenosine (v) women of childbearing age (vi) life expectancy <3 months (vii) previous intracranial haemorrhage and (viii) use of oral anticoagulation.

Procedural Details

PPCI was performed via radial artery route as default, with operators' discretion to switch to femoral route if required. All patients were pre-loaded with Aspirin 300mg and a P2Y12 inhibitor (Clopidogrel 600 mg or Ticagrelor 180mg). Patients were anticoagulated with a heparin bolus (70–100 U/kg) after arterial sheath insertion to achieve an activated clotting time (ACT) >250 s throughout the procedure. Glycoprotein IIb/IIIa inhibitors were given at the operator's discretion. Iopromide (Ultravist, Bayer HealthCare Pharmaceuticals, Leverkusen, Germany) was used as the contrast agent for all cases.

Modified Rentrop Collateral Score

0 – no collateral vessels; 1 – filling of side branches of the occluded artery via collateral channels without visualization of the epicardial segment; 2 – partial filling of the epicardial segment via collateral channels; 3 – complete filling of the epicardial segment of the occluded IRA via collateral channels.

CMR Protocol

CMR studies were performed using a 1.5 T CMR scanner (Magnetom Avanto, Siemens AG, Erlangen, Germany) within 24-72 h of PPCI. Image stacks were acquired using standard protocols. Both 'early' (following first pass perfusion) and 'late' (5–10 min later) enhancement images were acquired using gadolinium (Gadovist, Bayer Pharma AG, Berlin) at 0.2 mL/kg. Hypo-enhanced areas on late GAD enhancement were identified as areas representing microvascular obstruction (MVO). CMR data was analysed offline using semi-automated CMR42 software (Circle Cardiovascular Imaging, Alberta, Canada). Late GAD evidence of MVO was recorded as a binary measurement, and the MVO was quantified as percentage of LV mass.

Parameter Estimates

Grp[a]		B	Std. Error	Wald	df	Sig.	Exp(B)	95% Confidence Interval for Exp(B)	
								Lower Bound	Upper Bound
1.00	Intercept	-5.501	3.252	2.862	1	.091			
	Age	.067	.040	2.776	1	.096	1.069	.988	1.156
	Diabetes	-.840	.837	1.008	1	.315	.432	.084	2.226
	Smoking	-1.071	.740	2.098	1	.147	.343	.080	1.460
	Hypertension	.224	.754	.088	1	.767	1.251	.286	5.477
	Previous MI	1.182	1.135	1.085	1	.298	3.261	.353	30.162
	Hypercholestrolemia	.710	1.053	.455	1	.500	2.034	.258	16.012
	Statin use	-1.072	.905	1.403	1	.236	.342	.058	2.017
	Male	.872	1.046	.694	1	.405	2.391	.308	18.583

a. The reference category is: 2.00.

Multinomial Logistic Regression of baseline characteristics of patients with coronary steal (Group 1) vs. patients with no evidence of coronary steal (Group 2) as reference.
